# Enhanced Durability of Cellulose-Reinforced PVA-SA Beads for Long-Term Quorum Quenching Applications in Membrane Bioreactors

**DOI:** 10.3390/gels12060480

**Published:** 2026-05-30

**Authors:** Noman Sohail, Thomas Fischer, Marion Martienssen

**Affiliations:** 1 Chair of Biotechnology of Water Treatment, Brandenburg University of Technology Cottbus-Senftenberg, Siemens-Halske-Ring 8, 03046 Cottbus, Germany; marion.martienssen@b-tu.de; 2 Central Analytical Laboratory, Brandenburg University of Technology Cottbus-Senftenberg, Siemens-Halske-Ring 8, 03046 Cottbus, Germany; thomas.fischer@b-tu.de

**Keywords:** membrane bioreactor (MBR), quorum sensing (QS), quorum quenching (QQ), polyvinyl alcohol and sodium alginate (PVA-SA), CEBs (cell entrapping beads)

## Abstract

The long-term application of immobilized quorum quenching (QQ) bacteria requires carrier materials with sufficient mechanical stability and durability across various operating conditions. This study aims to enhance the durability and stability of polyvinyl alcohol (PVA) beads and to evaluate their performance for long-term operation. The beads were synthesized using two PVA brands with different molecular weights (MWs), and the effect of cross-linking conditions and reagent purity on bead stability was also investigated. Primarily, their physical strength was evaluated under centrifugal forces. Additionally, polyvinyl alcohol and sodium alginate (PVA-SA) beads were incorporated with cellulose to enhance their strength. The structural and chemical characteristics of the beads were examined using scanning electron microscopy (SEM) and Fourier-transform infrared spectroscopy (FTIR). The results showed that PVA 100 kDa beads withstood centrifugal forces up to 11,000 rpm without breakage, whereas lower MW (PVA 85 kDa) beads failed at 5000 rpm. Bead quality was critically sensitive to calcium chloride purity, as impurities and reduced Ca^2+^ availability caused poor crosslinking and structural collapse. The results revealed that PVA 100 kDa increases the number of polymer chain entanglements and intermolecular interactions, which enhance the structural integrity. Bead quality is strongly influenced by the purity of calcium chloride in the crosslinking solution, as well as by the solution pH. SEM analysis showed that cellulose-incorporating beads exhibited a denser and more uniform pore structure, with median equivalent pore diameters reduced from 50 µm (PVA-SA) to 22.4 µm upon cellulose incorporation, while maintaining sufficient porosity for nutrient diffusion. Similarly, FTIR analysis confirmed that cellulose was successfully integrated, with increased hydroxyl interactions and modified C–O vibrational characteristics, indicating strong hydrogen bonding within the composite matrix. Principal component analysis (PCA) confirmed that hydroxyl interactions and C–O vibrational modes are the main contributors to spectral variation, indicating that cellulose acts as a structural modifier in the PVA-SA network. These results demonstrate the effectiveness of this strategy in designing durable PVA-SA-cellulose based composite beads for long-term QQ applications.

## 1. Introduction

Membrane biofouling is a major challenge in implementing the membrane bioreactor (MBR) systems, as it hinders their development and large-scale applications [1,2]. Fouling is caused by the undesired accumulation of microorganisms and the release of extracellular polymeric substances (EPS) on the membrane surface, leading to reduced permeate flux, increased operational costs, and decreased membrane lifespan [3]. Initially, microorganisms secrete EPS, which facilitate the attachment and accumulation of microbial flocs and promote biofilm formation. EPS consists of diverse components, including proteins, polysaccharides, and humic substances [4]. These substances block membrane pores from both the internal and external sides, ultimately resulting in the formation of a dense cake layer on the membrane surface [5]. Therefore, it is important to understand the microbial behaviour involved in EPS regulation and synthesis, as well as in biofilm formation, to develop effective biofouling control strategies.

The microbial mechanism involved in biofilm development is known as quorum sensing (QS). In this process, bacteria produce autoinducers (AIs) for intercellular cell-to-cell communication. Acyl homoserine lactones (AHLs) are one of the main types of AIs used by Gram-negative bacteria and play a key role in regulating collective behaviours such as biofilm formation. When the concentration of AHL molecules reaches a critical threshold, they activate QS-regulated gene expression in a coordinated manner [6,7]. Quorum quenching (QQ) is a promising technique for mitigating biofouling, as it degrades AHL molecules involved in QS [8,9]. By disrupting QS, QQ inhibits EPS secretion, a key contributor to biofouling [10], and it has also been shown not to adversely affect treatment performance [11].

The practical applications of QQ represent a major research focus, particularly the encapsulation of enzymes and bacteria in various carrier materials [12]. Researchers have investigated various QQ media to enhance the efficiency of QQ bacteria. These media can exist in different shapes, such as vessels, beads, cylinders, and sheets. In early studies, QQ bacteria were entrapped in a non-free-moving polymeric vessel. However, this approach proved ineffective due to limited nutrient transfer within the vessel, thereby affecting bacterial growth and ultimately reducing QQ performance [13,14]. The use of beads for QQ cell entrapment proved to be more effective. This effectiveness arises from nutrients being transferred through the porous beads and from their spherical shape, which promotes physical interaction with the membrane, thereby providing a washing effect and reducing membrane fouling [15].

Polyvinyl alcohol-sodium alginate beads (PVA-SA) were found to be more stable than pure alginate beads, owing to their superior mechanical and chemical stability as well as their economic affordability for the immobilization of QQ bacteria [16,17]. Such immobilization media can protect QQ bacteria from bacterial competition present in sludge and from harsh environments, such as toxic substances found in industrial and sewage wastewater [18]. Beads can be prepared from different polymers, including Polyurethane, agarose, collagen, alginate and polyacrylamide. However, these materials often exhibit limitations, such as high cost, low structural integrity and poor durability [19].

Long-term MBR operational performance and fouling mitigation for 114 days have been previously reported by Sohail et al. [20]. Therefore, this study aims to prepare high-quality beads with enhanced stability and durability by strengthening the beads for the immobilization of QQ bacteria, particularly for long-term operations in MBR. Despite extensive research, existing studies have not sufficiently addressed the development of beads with improved durability and stability [21]. To address this gap, two types of PVA with different molecular weights (MW) were used. Although the PVA beads method has been described in previous studies [22], that approach was brand-specific; for instance, when calcium chloride from a different supplier was used, the resulting beads exhibited poor stability. Accordingly, this study systematically investigates calcium chloride of different purities and examines their effects on bead formation. Furthermore, to improve bead durability, cellulose was incorporated into the bead formulation, which, to the best of our knowledge, has not been previously investigated in QQ bead systems. The novelty of this study lies in addressing the major limitations of previously reported QQ beads, including insufficient mechanical durability, instability during long-term MBR operation, and poor reproducibility of bead synthesis caused by variations in crosslinking chemicals, particularly different types of calcium chloride. In particular, cellulose has not been previously incorporated into PVA-based beads for QQ applications, making this a novel approach for enhancing bead performance and structural stability. Cellulose was selected because its fibrous structure can reinforce the polymeric network, thereby improving bead integrity, stability, and resistance to deformation during prolonged operation. In addition, this work advances existing PVA-SA QQ bead studies by systematically evaluating the effects of different crosslinking conditions and reagent types on bead quality and long-term durability. The synthesized beads were subsequently evaluated to assess their stability and durability for potential long-term application in MBR systems.

## 2. Results and Discussion

### 2.1. Characterization and Evaluation of PVA-SA Beads

#### 2.1.1. Structural Stability of the Beads Under Different MW and Cross-Linking Conditions

**Figure 1:**
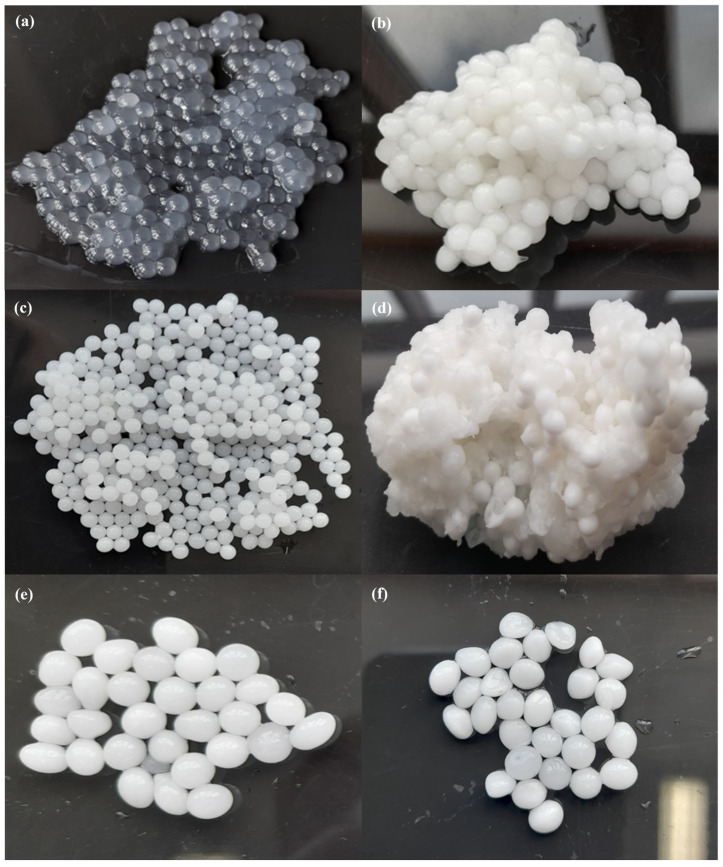
Structural comparison of PVA-SA beads under different preparation and centrifugal force. (**a**) PVA 85 kDa at a PVA-SA ratio of 8:1, (**b**) PVA 85 kDa at a PVA-SA ratio of 8:2, (**c**) PVA 100 kDa at a PVA-SA ratio of 8:1, (**d**) PVA 100 kDa at a PVA-SA ratio of 8:1 using (CaCl_2_ 97% purity). (**e**) PVA 100 kDa deformed by 10% at 12,000 rpm. (**f**) PVA 100 kDa deformed by 20% at 13,000 rpm.

This study presents a comparison of the structures and durability of the beads made of two different PVA brands with varying MW. The PVA-SA beads were spherical in shape, with an average diameter of 4 mm, and remained suspended in the reactor due to aeration. The suspension behavior of the beads is advantageous for MBR applications because, due to air scouring effects, the beads physically interact with the membrane surface, helping to minimize biofouling. This dynamic interaction enhances membrane surface cleaning, reduces the accumulation of foulants, and contributes to maintaining stable filtration performance [23]. Figure 1a shows beads synthesized using PVA 85 kDa at a PVA-SA-ratio of 8:1. These beads exhibited a soft texture, lacking mechanical strength. The reduced rigidity suggests insufficient intermolecular interactions within the polymer network, resulting in a low MW of PVA, which consequently leads to weak structural integrity and poor mechanical strength of the beads [24].

Figure 1b shows beads with the same MW (PVA 85 kDa) but with an increased SA content (PVA-SA) 8:2. The beads appeared more cohesive and still mechanically weak and structurally unstable, with irregular shapes and partial deformation. This observation indicates that simply increasing the alginate proportion does not necessarily enhance bead stability when the polymer chain length of PVA remains relatively low, as the overall structural integrity of the matrix still depends strongly on polymer chain entanglement and network formation. The beads in Figure 1c had a higher MW (PVA 100 kDa) and were prepared with an 8:1 ratio. These beads were smooth and round, and showed a compact and uniform shape, which apparently looked more stable than others. The improved morphology indicates the formation of a more cohesive polymeric matrix capable of resisting deformation during gelation and handling. This indicates that a higher MW increases the number of polymer chain entanglements and intermolecular interactions, which enhances the formation and stability of a stronger three-dimensional network during cross-linking. This leads to denser cross-linking and greater mechanical stability in the final beads, which is consistent with some previous studies related to the stability of beads [25,26].

Figure 1d demonstrates that the higher PVA 100 kDa with a similar ratio (8:1) was compromised when using lower-purity calcium chloride (Thermo Scientific, Ca^2+^ purity 97%) in the first cross-link solution. Approximately 70% of the beads dissolved when taken out of the second cross-linking solution. This drastic loss of structural integrity highlights the sensitivity of the ionic gelation process to the availability of free Ca^2+^ ions during bead formation. This was due to reduced Ca^2+^ availability from impurities and/or residual moisture. Impurities (other metal salts adsorbed) can readily compete with calcium ion (Ca^2+^) for the binding sites on the alginate chains, which can cause ineffective ionic cross-linking. Such competitive interactions disrupt the formation of the well-known “egg-box” structure responsible for alginate gel stabilization. On the other hand, residual moisture impurities can hydrolyze the calcium chloride, so the concentration of free calcium ions is reduced for the ionic cross-linking [27]. In contrast, high-purity anhydrous CaCl_2_ Merck (Darmstadt, Germany), produced stable beads, emphasizing the importance of reagent quality in crosslinking efficiency (Figure 1c). These findings underline that the higher MW of PVA affects the durability of the beads, and the purity of the reagents plays an important role in determining bead stability, which should be carefully controlled during the preparation of immobilization beads to ensure consistent structural integrity and long-term performance.

#### 2.1.2. Influence of pH on Cross-Linking

The results revealed how the MW of PVA influences the stability of the beads. Similarly, SA also plays an important role in achieving high-quality beads. SA is a naturally occurring anionic polysaccharide, derived from the kelp of bacteria or brown algae. Its molecular structure consists of β-d-mannuronic acid (M) and α-l-guluronic acid (G), interconnected by (1 → 4) bonds [28]. The stability of alginate-based beads is strongly governed by the protonation state of its carboxyl (–COO^−^) groups, which depends on the pH of the cross-linking solution. Since the pKa of sodium alginate lies around 3.3–3.6 [29], solutions with pH below this range promote protonation (–COOH) of the polymer chains. At pH 3.07 (boric acid + CaCl_2_, 97% purity), a significant fraction of the carboxyl groups becomes protonated, reducing the availability of negatively charged sites required for ionic cross-linking with Ca^2+^ ions. As observed in Figure 1d, the gel network is weakened, leading to partial dissolution of the beads after removal from the solution. These findings are consistent with previous studies [30].

When the pH was slightly increased to 3.2 using boric acid + CaCl_2_ anhydrous granules, the degree of protonation decreased marginally, allowing more –COO^−^ groups to interact with Ca^2+^ ions and form the characteristic “egg-box” structure. This resulted in beads that retained their shape, although they remained mechanically soft, indicating that cross-linking was present but not sufficiently strong. This pH is close to the pKa, which means there is a fine balance between protonated and deprotonated states, which restricts optimal network formation. The results align with the previous findings [31].

At a lower pH of 2.9 (boric acid + CaCl_2_·2H_2_O 99.5%), improved bead formation was observed, as shown in Figure 1c, which may seem counterintuitive given increased protonation. However, this behavior can be attributed to enhanced Ca^2+^ availability and controlled gelation kinetics, where rapid ionic interactions and possible secondary stabilization effects (e.g., hydrogen bonding or borate interactions) contribute to forming a more compact and stable gel network. The results align with those of the previous study; however, despite higher protonation at lower pH, the overall cross-linking efficiency and bead integrity are influenced by a combination of pH, calcium ion release, and salt form, leading to improved bead stability under these conditions [32].

#### 2.1.3. Physical Strength Under Centrifugal Force

**Figure 2:**
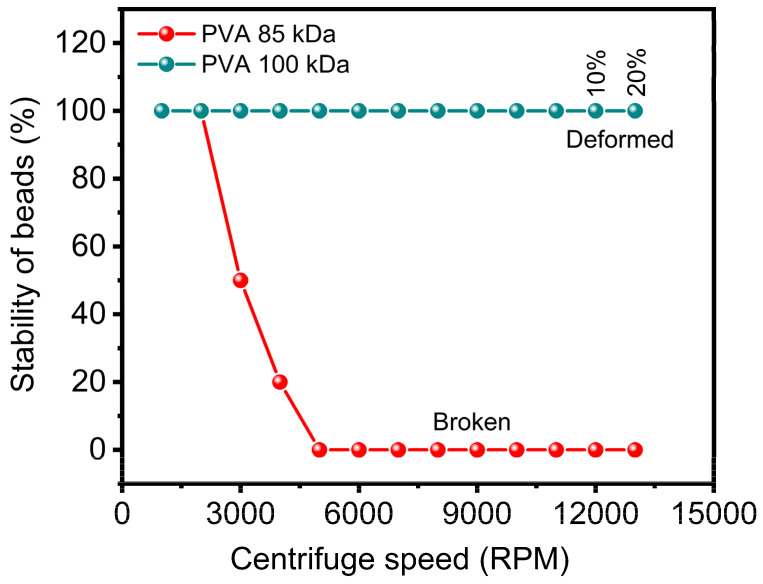
Comparison of the physical strength of PVA beads with two different MWs, illustrating the effect of PVA (MW) on bead mechanical strength.

For longer MBR operation, bead stability is a critical factor for bacterial immobilization, as unstable beads not only require frequent replacement but also compromise QQ bacterial performance and overall economic viability [26]. For this purpose, bead stability was analyzed under higher centrifugal force. Figure 2 illustrates the physical strength of the beads, demonstrating their stability up to 2000 rpm. However, at 3000 rpm, PVA beads with a MW (PVA 85 kDa) exhibited 50% breakage, and at 5000 rpm, nearly all beads were destroyed, indicating that they were not structurally stable. To enhance stability, PVA beads with a MW (PVA 100 kDa) were further tested. The results revealed that the beads remained intact up to 11,000 rpm. At 12,000 and 13,000 rpm, approximately 10% and 20% of the beads, respectively, deformed into an egg-like shape (Figure 1e,f) rather than breaking. These results suggest that PVA beads with a PVA 100 kDa exhibit significantly higher stability under higher centrifugal force.

To produce mechanically stable and structurally uniform beads, the comparative analysis was done while using two different PVAs. Good-quality beads maintain their shape and size even under shear stress, which is important for ensuring the long-term effectiveness of bacterial immobilization. Results of this study show that the low MW (PVA 85 kDa) produced unstable beads. Although the concentration of SA was increased, no significant improvement in bead stability was observed, suggesting that the lower molecular weight of PVA limits structural integrity and effective network formation irrespective of SA content. Instead of improving the structure, the beads often exhibited irregular shapes and partial deformation, suggesting that merely adjusting the SA concentration is insufficient to compensate for the structural weakness associated with shorter polymer chain lengths. On the other hand, beads synthesized with a higher MW (PVA 100 kDa) showed superior structural stability [33]. It was also observed that a weak cross-link solution also affected the beads. This is because insufficient cross-linking leads to a less interconnected polymer network, resulting in beads with lower mechanical strength and a higher chance of breaking and deformation. The physical strength of the beads was determined under increasing centrifugal forces, which provided additional validation of structural differences. Notably, the higher MW of PVA beads demonstrated not only resistance to destruction at elevated rpm but also exhibited less deformation rather than breaking, which is important for the immobilization of bacteria inside the beads during long-term operation. The enhanced stability of beads can be ascribed to a higher MW of PVA, which promotes intermolecular interactions. These two factors collectively increase the stability of beads under higher mechanical stress [24].

### 2.2. Bacterial Survival Inside the Beads over Time

**Figure 3:**
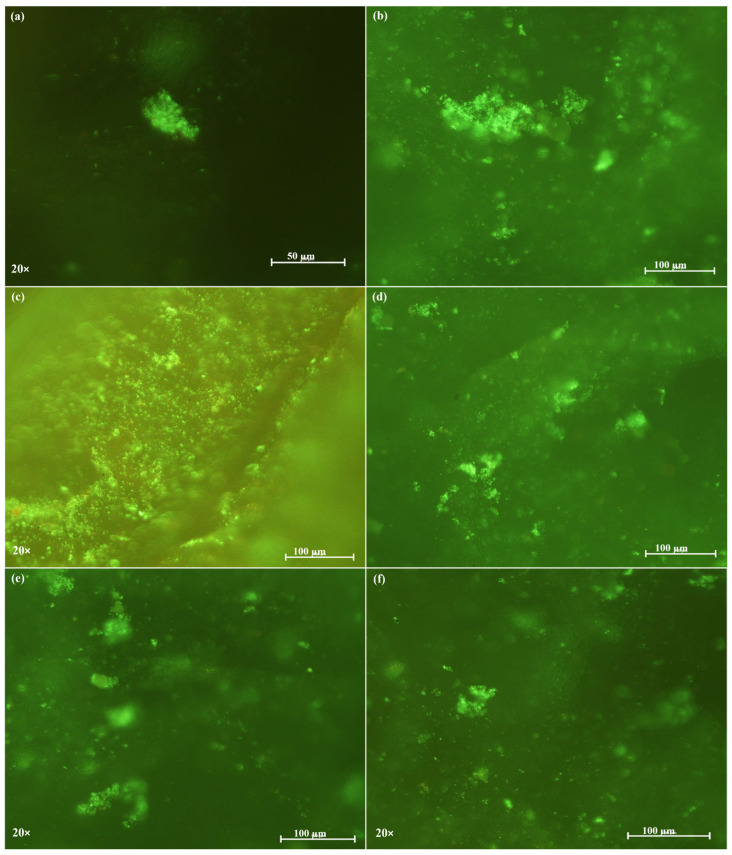
Fluorescent Microscopy images showing the visibility of bacterial cells encapsulated within the beads over time, namely (**a**) week 2, (**b**) week 4, (**c**) week 6, (**d**) week 8, (**e**) week 10, and (**f**) week 12, demonstrating bacterial proliferation within the beads over the incubation period.

In long-term MBR operation, the survival test indicated that the method for making CEBs worked well to protect bacteria from harsh environmental conditions over time. The survival of bacteria encapsulated within the beads was confirmed using fluorescence microscopy. The cross-section of the bead was examined under fluorescence microscopy, where green clusters, stained by SYTO 9, indicated live bacteria, while red cells, stained by propidium iodide, represented dead cells. Figure 3a shows the initial stage of the CEBs, where only a few bacteria were observed. This indicates that the QQ bacteria were successfully entrapped inside the beads. However, Figure 3b–f reveal an increasing number of bacterial colonies inside the beads over time. This gradual increase in bacterial clusters suggests that the encapsulated cells not only survived but also remained metabolically active and capable of proliferation within the bead during operation. The results demonstrate that the beads protect the bacteria from the harsh environment through encapsulation and also support the long-term viability of the bacteria as they proliferate in bioreactors. This protective effect is possibly due to the polymeric hydrogel network, which acts as a physical barrier against hydrodynamic shear stress, toxic compounds, and microbial competition present in the mixed liquor of the MBR system. Simultaneously, the porous nature of the beads facilitates the diffusion of nutrients and oxygen, thereby establishing a suitable environment for their survival and activity [34]. These findings are significant for biofouling control, as they demonstrate that QQ bacteria remain viable and active over extended periods within the reactor. The sustained presence of these bacteria is essential for disrupting the QS pathway and reducing biofilm formation on membrane surfaces. Moreover, the observed proliferation of bacteria within the beads suggests that the encapsulated system can function as a biological reservoir, thereby maintaining a stable microbial population throughout the operation. The effectiveness of this approach suggests that CEBs can serve as a promising strategy for long-term biofouling mitigation in MBR, offering a sustainable and efficient alternative to conventional antifouling methods.

### 2.3. Freeze-Drying Effects on Bead Structure

**Figure 4:**
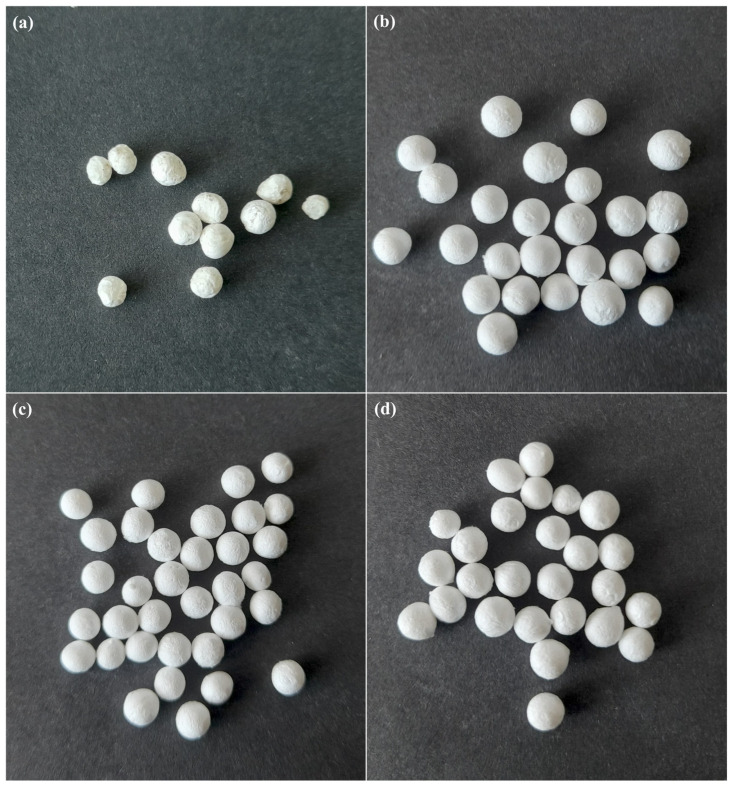
Morphological characteristics of the beads after freeze-drying: (**a**) PVA-SA beads prepared with granules of CaCl2, (**b**) PVA-SA beads, (**c**) PVA-SA-Cellulose beads containing 0.5 g cellulose, and (**d**) PVA-SA-Cellulose beads containing 1 g cellulose.

A freeze-drying test was applied to the beads to remove water content and to preserve the samples for further structural characterization, including SEM and FTIR analysis. Figure 4a shows that the bead structure was destroyed after the freeze-drying process. Although the beads appeared intact and visually similar to the other beads before being placed in the freeze dryer, the final morphology changed markedly after drying. The beads collapsed and lost their spherical structure, indicating weak structural integrity. This observation suggests insufficient or poor cross-linking during bead preparation, particularly in the first cross-linking step, where anhydrous granules of CaCl_2_ solution were used. The uneven distribution of CaCl_2_ may have resulted in inadequate ionic cross-linking, which weakened the bead matrix and caused structural collapse during freeze drying [35]. During freeze-drying, the removal of ice crystals generates internal stresses within the hydrogel network, and if the polymer matrix is not sufficiently cross-linked, the porous structure cannot bear this stress, leading to structural collapse. On the other hand, the remaining three types of beads (Figure 4b–d) maintained their shape and size after freeze-drying. Although these beads were prepared using different materials and concentrations, their structural integrity was preserved. Figure 4b corresponds to PVA-SA beads, Figure 4c represents PVA-SA-Cellulose (0.5 g), and Figure 4d shows PVA-SA-Cellulose (1 g) of cellulose concentration. The reason for the preserved morphology is that their cross-linking was stronger, in which high-purity CaCl_2_·2H_2_O (99.5%) was used as the cross-linking agent. Because of that, their shape and structural stability were maintained [36]. Secondly, Figure 4c,d show the incorporation of PVA-SA with cellulose, which enhanced the mechanical stability of the polymer network through additional hydrogen-bonding interactions within the matrix. It was also observed that when the beads were dried at room temperature, their shape was distorted. However, after being re-immersed in water, they restored their shape, and a similar recovery behavior was also observed for freeze-dried beads that initially lost their shape, but regained it upon rehydration. These results also align with previously reported studies [33]. This behavior is typical of hydrogel materials, which exhibit reversible swelling–shrinking due to their hydrophilic polymer networks. The chains of polymers swell and return to their initial shape when they are rehydrated [37].

### 2.4. Structural Morphology of the Beads Using Scanning Electron Microscopy

**Figure 5:**
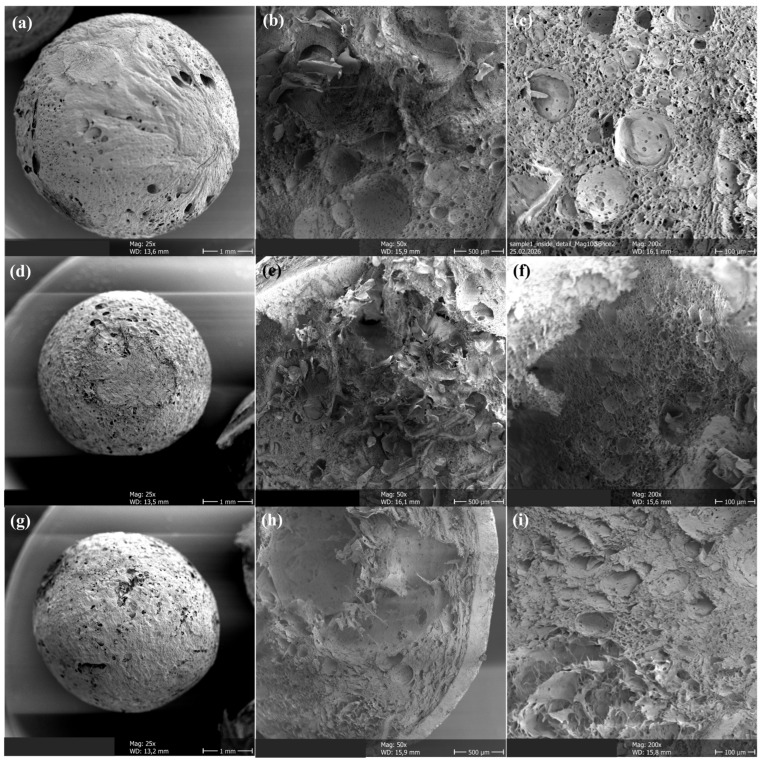
SEM images showing the external morphology and internal cross-sectional structures of the beads at different magnifications. (**a**–**c**) PVA-SA beads: (**a**) external surface at 25×, (**b**) cross-sectional views at 50× and (**c**) cross-sectional views at 200×. (**d**–**f**) PVA-SA-Cellulose (0.5 g) beads: (**d**) external surface at 25×, (**e**) cross-sectional views at 50× and (**f**) cross-sectional views at 200×. (**g**–**i**) PVA-SA-Cellulose (1 g) beads: (**g**) external surface at 25×, (**h**) cross-sectional views at 50× and (**i**) cross-sectional views at 200×.

Scanning electron microscopy (SEM) was used to examine the external and internal structure of the beads. The outer surface of the beads was evaluated based on surface smoothness, roughness, and the presence and distribution of pores. The internal structure of the beads was differentiated primarily according to pore size and pore distribution across the three different samples, which is particularly important because the entrapped QQ bacteria rely on these pores for the diffusion of nutrients and their metabolic activity [33]. Figure 5a shows an uneven surface with visible flow-like patterns and moderate roughness. Several large and irregularly shaped pores are present on the surface. These larger voids may act as stress concentrations, which can negatively affect the mechanical stability of the beads [38]. In contrast, Figure 5d exhibits a rougher surface with small and uniformly distributed micropores. The presence of smaller, well-distributed pores indicates a more controlled porous structure, which can contribute to improved mechanical strength. Similarly, Figure 5g demonstrates beads with a comparatively smoother and more compact surface with smaller visible pores, suggesting improved structural consolidation during bead formation. Since the mechanical strength and durability of the beads are strongly influenced by surface integrity and porosity, the lower porosity and improved surface compactness observed in Figure 5g suggest better mechanical stability compared with the other two samples. However, while denser structures improve mechanical durability, an appropriate pore network is still essential to allow mass transfer of nutrients and metabolites to sustain the activity of the entrapped QQ bacteria. Therefore, based on the SEM observations, the PVA-SA-Cellulose (1 g) beads exhibit higher structural strength and durability due to their denser and more compact surface structure, while maintaining sufficient porosity for nutrient diffusion.

Figure 5b reveals the internal structure of the bead, in which large pores and irregular cavities were observed throughout the bead matrix. Many pores appear elongated, indicating a highly porous and loosely packed internal network. The presence of large macropores and high porosity may facilitate efficient mass transfer and nutrient diffusion to the immobilized bacteria. However, large pores and voids may also reduce the structural integrity and weaken the mechanical stability of the beads, which is consistent with the structural deterioration observed during long-term MBR operation. Figure 5e shows that the size of the pores is distributed within a rough and irregular matrix. However, compared to the previous sample, the pores are smaller. There are also some interconnected channels and fragments. Overall, the image suggests that the pores and interconnected structure are sufficient to allow nutrient diffusion, while the matrix remains structurally stronger and more stable than Figure 5b. The internal structure of Figure 5h is comparatively denser and more compact, with smaller pores visible within the matrix. The pores are more uniformly distributed and significantly smaller, indicating lower internal porosity. This dense microstructure suggests improved structural consolidation during bead formation, which enhances the mechanical strength and durability of the beads. The presence of micro-scale pores still allows diffusion of nutrients and metabolic products, which is essential for maintaining the activity of the entrapped QQ bacteria.

The same sample was examined at higher resolution (200×) to obtain a clearer view of the previously observed structural features, which further confirmed the earlier findings. As observed, Figure 5c exhibits a highly porous and heterogeneous structure, containing numerous large cavities and irregularly distributed pores, indicating high internal porosity but relatively weak structural consolidation. In contrast, Figure 5f shows a much denser and more compact matrix with smaller and more uniformly distributed pores, suggesting improved structural integrity and potentially higher mechanical stability. Figure 5i presents moderate porosity and irregular pore distribution, including some larger voids and partially collapsed regions within the matrix. Overall, it maintains a dense and well-consolidated structure, which reflects improved bead integrity and structural stability. The balanced microstructure, combining high structural integrity with sufficient micro-porosity, facilitates both mechanical stability and effective mass transfer within the beads. Based on the observed pore morphology from SEM analysis, cellulose incorporation is expected to influence nutrient diffusion pathways and microbial microenvironments through modifications in internal structure and pore connectivity. Consequently, this structure is advantageous for long-term application, as it helps preserve bead integrity while sustaining the metabolic activity of the immobilized QQ bacteria. Table 1 presents a comparison of the morphological characteristics of the bead samples.

**Figure 6:**
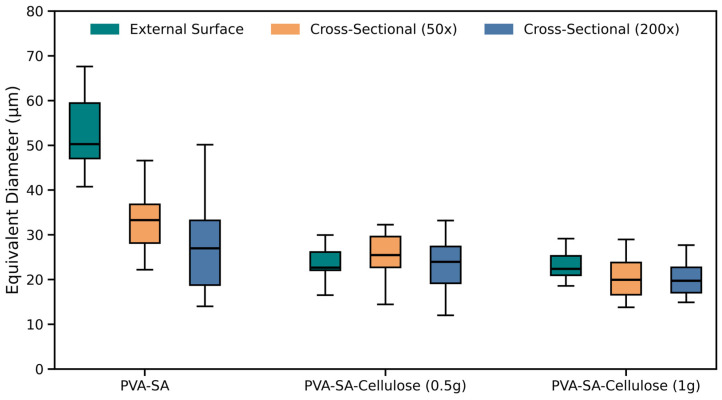
Equivalent pore diameter (µm) of external (25×) and cross-sectional regions (50× and 200×) of PVA-SA and PVA-SA–cellulose (0.5 g) and PVA-SA-cellulose (1 g).

The statistical analysis of the pore size distribution was conducted for three samples: PVA-SA and PVA-SA-cellulose composite beads containing 0.5 g and 1 g of cellulose. Pore size was determined using the estimated surface area (µm^2^) and equivalent diameter (µm) based on both the external surface and internal cross-sectional structure at magnifications 50× and 200×. Table 2 illustrates the mean ± standard deviation (SD) of the pore size distribution across the three different samples, while representative pore size measurement locations in the corresponding SEM images are highlighted with circles in Supplementary Figure S1 for clarity. Figure 6 compares the beads by equivalent diameter (µm). The external surface of PVA-SA exhibits the largest median of 50 µm. Incorporating 0.5 g of cellulose reduced the median to 22.7 µm, indicating a smaller and more uniform pore structure. Further increasing cellulose to 1 g resulted in a slight additional reduction to 22.4 µm, with an even narrower distribution. Similarly, the cross-sectional (50×) view of PVA-SA shows the highest median of 33.3 µm. The addition of 0.5 and 1 g cellulose reduced the median to 25.5 and 19.9 µm, respectively. A similar trend was observed in the cross-sectional (200×) view at higher magnification, where the median pore size of PVA-SA was 26.9 µm, which decreased to 23.9 µm with 0.5 g cellulose and to 19.7 µm with 1 g cellulose. These findings highlight that the cellulose incorporation significantly reduces pore size and refines the overall bead structure in both the external and internal surfaces.

### 2.5. ATR-FTIR Spectroscopy

**Figure 7:**
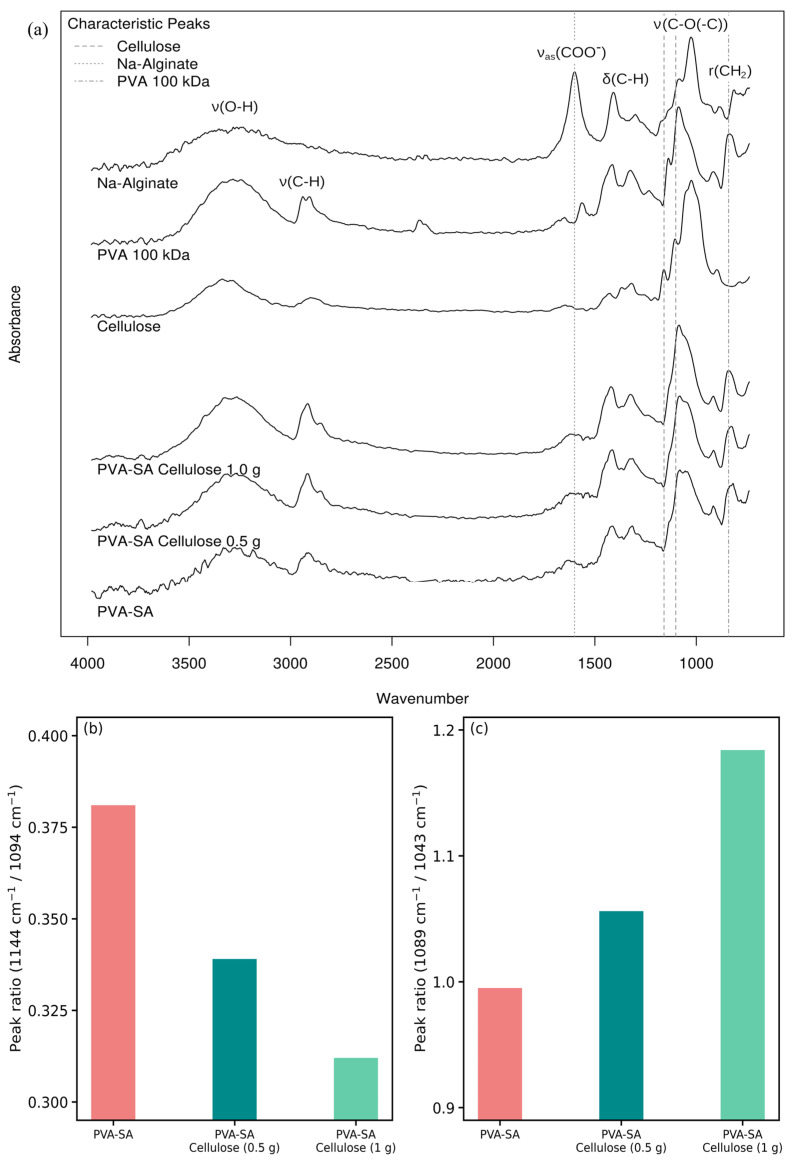
(**a**) Normalized FTIR Spectra of the pure components (PVA, SA and cellulose) and prepared composites, including PVA-SA, PVA-SA cellulose composite containing 0.5 g, and 1 g cellulose, with the characteristic peaks assigned to ν(O–H) stretching OH vibration, ν(C–H) stretching CH vibration, ν_as_(COO^−^) asymmetric stretching carboxylate vibration, δ(C–H) deformation CH vibration, δ(C-O(-C)) stretching C-O and C-O-C vibrations, r(CH_2_) rocking CH_2_ vibration. (**b**) FTIR structural indices of PVA-SA, PVA-SA-Cellulose composites. (**c**) Crystallinity index calculated from the absorbance ratio between 1144–1094 cm^−1^, and C–O stretching index calculated from the peak ratio 1089–1043 cm^−1^, illustrating the effect of increasing cellulose content on the structural organization and intermolecular interaction within the bead matrix.

The normalized Fourier transform infrared spectroscopy (FTIR) spectra were obtained by applying the Savitzky–Golay smoothing and standard normal variate (SNV) normalization algorithms to the raw data [39]. The recorded spectra allow identification of the main functional groups and evaluation of possible interactions within the polymer-cellulose chain. The spectra exhibit characteristic absorption bands corresponding to hydroxyl groups, aliphatic C–H stretching vibrations, and C–O stretching modes typical for alcohols and polysaccharides. The spectral features are consistent with the presence of PVA, SA and cellulose. Figure 7a illustrates the FTIR spectra in which the upper three spectra represent the reference spectra of the pure components (PVA, SA, and cellulose), while the lower spectra correspond to the synthesized composites. The dotted vertical lines indicate the characteristic peak positions used for comparison. All samples exhibit a broad absorption band in the region 3680–2980 cm^−1^, corresponding to O–H stretching vibrations originating from hydroxyl groups present in PVA, SA and cellulose [40]. For the composite spectra, the integrated peak area in this region for PVA-SA is 93.99. This represents the lowest integrated absorbance in the hydroxyl region, as expected, since the beads contain only hydroxyl groups from PVA and alginate. The integrated peak area reaches 112.6 upon the incorporation of cellulose. This increase is attributed to the addition of 0.5 g cellulose, indicating a higher contribution of hydroxyl groups and enhanced hydrogen bonding interactions within the composite matrix. Cellulose contains a high density of hydroxyl groups, which can interact with the hydroxyl groups of PVA and the functional groups of alginate through intermolecular hydrogen bonding, thereby increasing the overall O-H absorbance. When the cellulose content is further increased to 1 g, the integrated absorbance increases to 130.6.

The region between 2980 and 2700 cm^−1^ corresponds to aliphatic C–H stretching vibrations of –CH_2_– and –CH_3_ groups, which mainly originate from PVA. The integrated peak areas in this region were 20.21 for PVA-SA, 22.80 for PVA-SA-Cellulose (0.5 g), and 24.45 for PVA-SA-Cellulose (1 g). The difference between all samples is relatively small, which indicates that the polymer structure remains unchanged, with only a small difference after the incorporation of cellulose. The slight increase in peak area observed for the cellulose-containing samples may result from minor structural rearrangements within the polymer matrix. The 1000–1100 cm^−1^ region of the spectra is important for polymeric polysaccharides because it contains several C–O stretching vibrations. In this particular situation, the evaluation of the absolute intensity of the 1089 cm^−1^ band and the peak ratio between 1089 cm^−1^ and 1043 cm^−1^ was considered. This strategy is more appropriate for normalized spectra and helps to better distinguish between the samples on the basis of intensity variations. These two bands correspond to C–O stretching vibrations, but they originate from slightly different structural environments within the polymer and polysaccharide network. The band at 1043 cm^−1^ is also linked to polysaccharide C–O vibrations and therefore provides a useful internal reference. Cellulose typically shows a strong FTIR absorption near 1059 cm^−1^, which corresponds to the C–O stretching vibration of the glucopyranose ring. However, in this study, the most responsive peak appears near 1089 cm^−1^, rather than at the typical cellulose maximum, which is consistent with previous studies [41]. It is suggested that the vibrational features of cellulose were influenced by the surrounding polymer matrix. The FTIR results suggest that when cellulose is incorporated into the PVA-SA matrix, the hydroxyl stretching intensity is increased upon cellulose addition, indicating enhanced hydrogen bonding interactions within the composite system. Meanwhile, the relatively stable C–H stretching region suggests that the backbone structure of the polymer matrix is not significantly affected. The behavior of the C–O stretching region further indicates that cellulose is present in the polymer matrix through hydrogen bonding, rather than existing as a separate phase. These interactions lead to modified vibrational characteristics and could suggest partial confined cellulose structure within the PVA-SA network, which may influence the structural and mechanical properties of the resulting beads. However, these interpretations should be further supported by additional structural analyses such as XRD, DSC, or solid-state NMR.

To investigate further structural changes in the beads upon cellulose incorporation, peak ratios were derived from the spectra. Figure 7b represents the crystallinity index (CI), which was estimated using the ratio of the band at 1144 cm^−1^/1094 cm^−1^ [41,42]. The CI gradually reduced as the cellulose concentration (0.5) was incorporated, and with the further increase in cellulose (1 g), it reduced even more. The reduction in the CI shows that cellulose induces changes in the arrangement of the polymer chains within the PVA-SA matrix. The hydroxyl groups of cellulose can interact strongly with the hydroxyl groups of PVA and the functional groups of SA through intermolecular hydrogen bonding, leading to a rearrangement of the polymer network. Such interactions may reduce the regular packing of the polymer chains and consequently lower the apparent crystallinity detected by FTIR. Similarly, the decrease becomes more pronounced at higher cellulose concentration, indicating that increasing cellulose concentration enhances the disturbance of the original polymer chain organization. This behavior suggests that cellulose acts as a structural modifier within the matrix rather than simply forming a separate phase.

The C–O ratio was evaluated from the 1089 cm^−1^/1043 cm^−1^ bands, which are associated with C–O stretching vibrations characteristic of polysaccharide structures. Figure 7c shows the opposite trend compared to CI; as the cellulose content increased, the C–O ratio increased, with the lowest value observed in PVA-SA without cellulose. Both peaks correspond to C–O stretching vibrations, which are characteristic of alcohol and polysaccharide structures. The increased ratio of C–O, particularly with the incorporation of cellulose, suggests its confinement within the PVA-SA network, where its interaction modifies the polymer chain structure. Therefore, the 1089 cm^−1^ and 1043 cm^−1^ ratio provides a more reliable indicator of cellulose incorporation in normalized spectra than the evaluation of a single peak intensity. These results suggest that cellulose is well integrated within the PVA-SA matrix, where it interacts with the surrounding polymers through hydrogen bonding. Table 3 summarizes the FTIR peak assignments for PVA, SA, and cellulose composite beads, indicating the functional groups and suggesting interactions among the components in the synthesized bead matrix.

**Figure 8:**
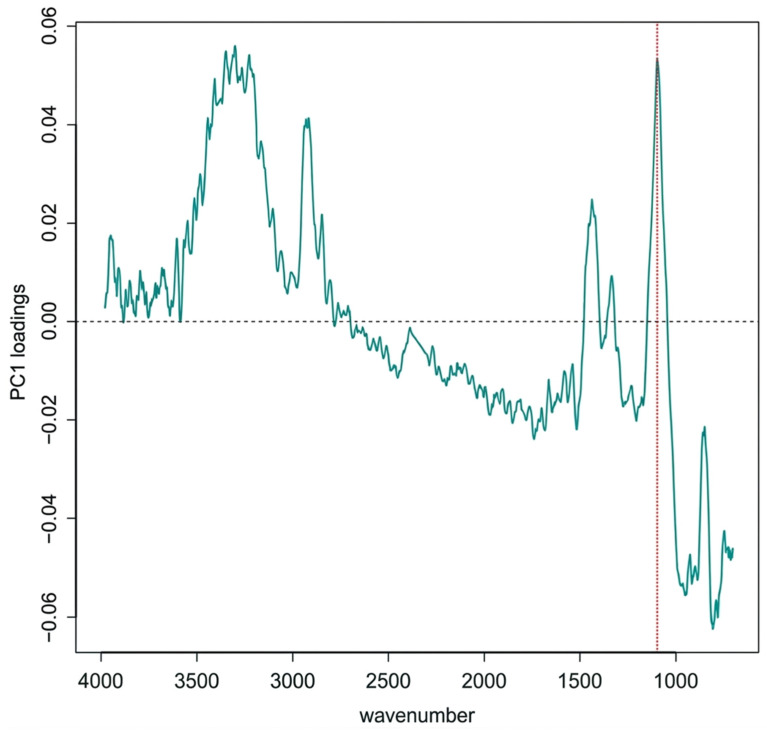
Principal component analysis (PC1 explaining 91.6% of total variance) of FTIR Spectra.

The principal component analysis (PCA) was applied to investigate the variation between different samples of the spectra. PCA is a multivariate statistical method that simplifies the complexity of the spectral datasets into a smaller number of orthogonal variables known as principal components (PCs), which capture the main sources of variance from the data [43]. The PC1 represents the direction of highest variance in the dataset and therefore describes the most significant spectral differences between the samples. Figure 8 illustrates the PC1 loading plot, indicating which spectral regions contribute most significantly to the variance among the samples. The most prominent regions in the spectra are associated with O–H stretching vibrations around 3600–3200 cm^−1^, C–H stretching vibrations near 2900 cm^−1^ and near 1450 cm^−1^, as well as a C–O stretching vibration at 1100 cm^−1^, which is characteristic of cellulose. These regions correspond to the functional groups that dominate the chemical structure of PVA, sodium alginate, and cellulose. The strong contributions from the hydroxyl region indicate that variations in hydrogen bonding interactions play a major role in distinguishing the samples. The incorporation of cellulose introduces additional hydroxyl groups, which can interact with PVA and alginate through intermolecular hydrogen bonding interaction, leading to changes in the intensity and shape of the O–H stretching band. Similarly, the loadings in the C–O stretching region suggest that the polysaccharide-related vibrational modes strongly influence the spectral differences between samples. This observation is consistent with the previously discussed 1089/1043 cm^−1^ peak intensity ratio, which increases with increasing cellulose content and reflects the growing contribution of cellulose within the bead matrix. Overall, the PCA demonstrates that the major spectral differences among the samples arise from changes in hydroxyl interactions and C–O vibrational modes, which are directly related to the progressive addition of cellulose and its structural interaction with the polymer network.

## 3. Conclusions

The study demonstrates that the bead stability was strongly dependent on the MW of PVA, while the purity of calcium chloride significantly affected bead formation, as impurities and reduced Ca^2+^ availability led to poor cross-linking and structural instability. Mechanical strength analysis showed that PVA 100 kDa beads maintained their shape even at higher centrifugal force and only showed 20% deformation at 13,000 rpm, indicating superior structural resistance compared to beads prepared with a lower MW of PVA. To further enhance bead stability, cellulose was incorporated into the PVA-SA matrix, resulting in a significant improvement in the bead structure and integrity. SEM analysis confirmed that PVA-SA-cellulose beads exhibited a smoother surface, a more uniform pore distribution and sufficient porosity for nutrient diffusion, which is essential for maintaining bacterial activity. In addition, cellulose incorporation reduced both external and cross-sectional pore diameters, indicating the formation of a more compact and stable network structure, as further supported by statistical analysis. FTIR analysis further confirmed the successful incorporation of cellulose into the PVA-SA matrix and revealed enhanced intermolecular interactions, particularly through hydrogen bonding, which contributed to improved network stability without altering the fundamental polymer structure. PCA of FTIR spectra accounted for 91.6% of total spectral variance via PC1, identifying hydroxyl and C–O stretching regions as the primary contributors to compositional differences among the bead formulations. Overall, these findings demonstrate that optimization of PVA properties and cellulose incorporation significantly improves bead durability and structural stability, highlighting the potential of these beads for long-term QQ applications in membrane bioreactors.

## 4. Materials and Methods

### 4.1. Chemicals

Two different brands of PVA (Thermo Scientific, Loughborough, UK) with a purity of 96.5% and an approximate MW of 85,000 g/mol, and PVA (UNI-Chem, Lahore, Pakistan) with a purity of 99% and an approximate MW of 100,000 g/mol, were used. Sodium alginate was purchased from Fisher Scientific (Loughborough, UK). Boric acid was purchased from AppliChem (Darmstadt, Germany), and three different brands of calcium chloride were used to achieve optimum cross-linking. Calcium chloride anhydrous (CaCl_2_) was purchased from Merck (Darmstadt, Germany); calcium chloride (97% dried powder) was purchased from Thermo Scientific (Kandel, Germany), for which the degree of hydration was not specified by the manufacturer (MW = 110.99 g/mol, consistent with the anhydrous form); and calcium chloride anhydrous granules and calcium chloride-dihydrate (99.5%) were purchased from Merck (Darmstadt, Germany). Sodium sulphate anhydrous (Na_2_SO_4_) was purchased from Fluka (Buchs, Switzerland). Cellulose was purchased from Sigma-Aldrich (Schnelldorf, Germany). The fluorescent dyes SYTO 9 and propidium iodide were both purchased from Invitrogen (Oregon, OR, USA).

### 4.2. MBR Plant Description

The MBR system was operated with a working volume of 4 L, a hydraulic retention time (HRT) of 5.1 h and a target mixed liquor suspended solids (MLSS) concentration of 8 g L^−1^. Real municipal wastewater was used in this study, which was collected from a local wastewater treatment plant. A hollow fiber polyvinylidene difluoride (PVDF) ultrafiltration membrane (pore size 0.1 μm, PHILOS, Gwangmyeong-si, Korea) was used for filtration. The transmembrane pressure (TMP) was measured by a SPER scientific data logging manometer (840099, Sper Scientific Limited Liability Company, San Bernardino, CA, USA). Two types of flat sheet membranes were also immersed in the MBR to evaluate biofilm formation. Polytetrafluoroethylene (PTFE) (Sartorius, Göttingen, Germany) and polysulfone (PS) (Alfa Laval Mid Europe GmbH, Hamburg, Germany) were used as 0.2 µm microfiltration membranes. These membranes were not operated for filtration, but were used to monitor biofilm development on the surface of the membranes [20].

### 4.3. Preparation of PVA-SA Beads

**Figure 9:**
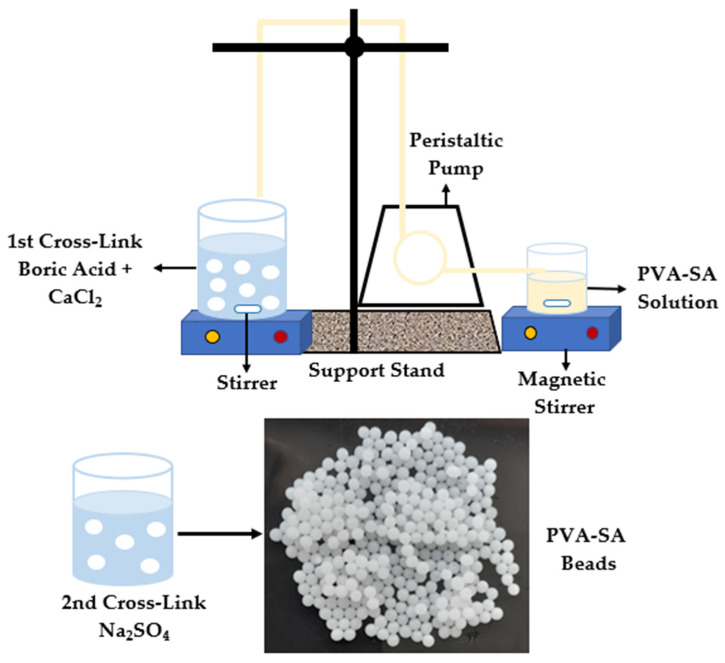
Schematic representation of the PVA-SA bead preparation process and the associated chemical crosslinking interactions involving borate ions with PVA hydroxyl groups and Ca^2+^ ions with alginate chains.

Two different PVA brands, with MW of PVA 85 kDa and PVA 100 kDa, were used in this study. The method described by Islam et al. [33] was used to produce PVA-SA beads. PVA and SA were mixed and dissolved in deionized water at concentrations of 0.08 g mL^−1^ and 0.01 g mL^−1^, respectively, using 8 g PVA and 1 g SA in a total volume of 100 mL of distilled water. The mixture was then placed in a drying oven at 105 °C for 4 h to obtain a completely homogeneous solution, which is important for producing high-quality beads with enhanced durability and mechanical strength. A schematic representation of the bead preparation method and the chemical crosslinking interactions is shown in Figure 9. The mixture was dripped through a peristaltic pump into the first cross-linking solution, consisting of boric acid (0.07 g mL^−1^) and calcium chloride anhydrous (0.04 g mL^−1^), corresponding to 7 g boric acid and 4 g calcium chloride dissolved in 100 mL of distilled water. The temperature of the first cross-linking solution was maintained at 35 to 40 °C for all experiments, regardless of the type of calcium chloride used. In the case of calcium chloride dihydrate, the concentration was slightly increased to an optimized value of 0.053 g mL^−1^. During the first crosslinking step, Ca^2+^ ions ionically crosslinked the alginate chains, while borate ions interacted with the hydroxyl groups of PVA, forming a stable three-dimensional polymeric network. The spherical beads formed were soaked in this solution for 30 min and then washed with deionized water. Subsequently, the beads were immersed in a second cross-linking solution containing sodium sulfate (0.07 g mL^−1^) for 2 h. Finally, the beads were washed again with deionized water and stored in deionized water at 4 °C. After preparation, the beads were evaluated using a centrifuge (UNIVERSAL 320R, Tuttlingen, Germany). The beads were placed in centrifuge tubes and rotated at different speeds, ranging from 1000 to 13,000 rpm for 10 min to check their mechanical strength.

### 4.4. Immobilization of QQ Bacteria

After the preparation of the vacant beads, a pure strain of the QQ bacterium, *Rhodococcus* sp. BH4 (Accession no. CP014941), was employed. The bacterial strain was first cultured on agar plates, and a single fresh colony was picked and transferred into 50 mL of sterilized R2A broth to establish the primary culture. The culture was incubated on an orbital shaker (Certomat MO II, Göttingen, Germany) at room temperature for 24 h. Then, a 450 mL secondary culture was prepared by inoculating the primary culture, followed by incubation on the shaker for another 24 h. After incubation, the bacterial suspension was collected by centrifugation at 5000 rpm for 10 min. The resulting bacterial pellet was then resuspended in 2 mL of deionized water and added to the PVA-SA solution. The mixture was homogenized thoroughly to achieve a uniform distribution of bacterial cells. The prepared suspension was subsequently utilized for the production of cell-entrapping beads (CEBs).

### 4.5. Survival of QQ Bacteria in Beads and Biofilm Assessment

Cell-entrapping beads were taken out of the reactor once a week. The beads were carefully sliced using a sterile scalpel to expose the internal structure without disrupting the embedded bacterial cells. The sliced sections were then examined using an epifluorescence microscope (ECLIPSE LV100, Nikon, Tokyo, Japan) to determine the bacteria’s survival and growth within the bead. Images were taken from the inside of the bead at high-resolution magnifications of 20× to clearly visualize the presence of bacteria. The samples were subjected to staining using the LIVE/DEAD assay (BacLight TM Bacterial Viability Kits, Invitrogen, Karlsruhe, Germany), which contains two fluorescent dyes. SYTO 9 stains green, indicating live bacteria, whereas propidium iodide stains red, indicating dead bacteria.

### 4.6. Synthesis of PVA-SA and Cellulose Beads

After the use of beads in long-term operation, it was realized that the beads required greater mechanical strength and durability. To improve these properties, cellulose was incorporated into the PVA-SA beads. Two cellulose concentrations (0.005 g mL^−1^ and 0.01 g mL^−1^) were used in combination with PVA-SA. The PVA-SA concentration and cross-linking solution concentration were kept the same as those described in Section 4.3.

### 4.7. Characterization of the Beads

To analyze the bead samples, PVA-SA and PVA-SA-Cellulose were frozen overnight and then freeze-dried (Alpha 1-4 LSCplus, Osterode am Harz, Germany) for 24 h. The samples were subsequently sputter-coated with gold (SCD 050, Wetzlar, Germany) for further observation. Afterwards, the sample was examined using scanning electron microscopy (SEM) (DSM 962, point electronic and Zeiss, Oberkochen, Germany) to analyze the surface morphology and internal structure of the beads. In addition, the dried beads were used for further analysis by Fourier transform infrared (ATR-FTIR) spectroscopy (PIKE Technologies, Madison, WI, USA; NICOLET 380, Waltham, MA, USA) at spectra ranging from 700 to 4000 cm^−1^. Two replicates were recorded for each sample, after which a Savitzky–Golay (SG) filter and SNV normalization were applied for data processing and interpretation.

### 4.8. Statistical Analysis

The statistical analysis of the SEM images was performed to determine the bead pore size distribution based on the estimated pore area (µm^2^) and equivalent diameter (µm), and the images were examined using NIS-Elements software (Nikon, Tokyo, Japan). OriginPro 2021 (OriginLab Corporation, Northampton, MA, USA) was used to generate the box plot. For the external surface of the bead, a total of (*n* = 10) sampling points were analysed. The internal (cross-sectional) measurements were carried out at two magnifications: 50× magnification (*n* = 20) and 200× magnification (*n* = 30). The mean ± standard deviation was calculated for both parameters to evaluate the average pore size and variability within the sample.

## Figures and Tables

**Table 1:** Comparison of three different types of beads.

Beads Morphology	Feature	PVA-SA	PVA-SA-Cellulose (0.5 g)	PVA-SA-Cellulose (1 g)
External Surface	Shape	Spherical	Spherical	Spherical
	Porosity	High (Macropores)	Moderate (well-distributed) Micropores	Smaller visible pores
	Roughness	High/Uneven	Moderate/Uniform	Low (Smooth)
Internal Cross-section	Pore distribution	Irregular	Highly interconnected	Smaller (uniformly distributed)

**Table 2:** Statistical analysis of the external and internal pore sizes of three different types of beads.

Beads Composition	Properties	External Surface No. of Count|Mean ± SD	Cross-sectional (50×) No. of Count|Mean ± SD	Cross-sectional (200×) No. of Count|Mean ± SD
PVA-SA	Estimated Area (µm^2^)	10/(22.17 ± 7.28) × 10^2^	20/(9.39 ± 4.91) × 10^2^	30/(7.6 ± 7.56) × 10^2^
	Equivalent Diameter (µm)	10/(5.24 ± 0.9) × 10^1^	20/(3.36 ± 0.84) × 10^1^	30/(2.86 ± 1.23) × 10^1^
PVA-SA-Cellulose (0.5 g)	Estimated Area (µm^2^)	10/(4.56 ± 1.83) × 10^2^	20/(6.22 ± 3.47) × 10^2^	30/(4.72 ±2.03) × 10^2^
	Equivalent Diameter (µm)	10/(2.36 ± 0.47) × 10^1^	20/(2.72 ± 0.73) × 10^1^	30/(2.39 ± 0.54) × 10^1^
PVA-SA-Cellulose (1 g)	Estimated Area (µm^2^)	10/(4.32 ± 1.28) × 10^2^	20/(3.48 ± 1.57) × 10^2^	30/(3.35 ±1.28) × 10^2^
	Equivalent Diameter (µm)	10/(2.32 ± 0.34) × 10^1^	20/(2.05 ± 0.47) × 10^1^	30/(2.03 ± 0.39) × 10^1^

Values represent mean ± standard deviation (External surface (25×): *n* = 10; Cross-sectional (50×): *n* = 20; Cross-sectional (200×): *n* = 30 points of sample).

**Table 3:** FTIR peak assignments for PVA, SA, and cellulose composites.

Spectral Region (cm^−1^)	Vibrational Assignment	Components
3680–2980	O–H stretching	Hydroxyl groups from PVA-SA
2980–2700	Aliphatic C–H stretching vibrations of –CH_2_– and –CH_3_ groups	Associated with PVA
1000–1100	C–O stretching	Alcohol and glycosidic groups
1089–1043	C–O stretching	Cellulose
1059	C–O stretching	Cellulose

## Data Availability

Data are contained within the article.

## Supplementary Materials

The following supporting information can be downloaded at https://www.mdpi.com/article/10.3390/gels12060480/s1. Figure S1: SEM images showing the external morphology and internal cross-sectional structures of the beads at different magnifications. (a–c) PVA-SA beads: (a) externally surface at 25×, (b) cross-sectional views at 50× and (c) cross-sectional views at 200×. (d–f) PVA-SA-Cellulose (0.5 g) beads: (d) externally surface at 25×, (e) cross-sectional views at 50× and (c) cross-sectional views at 200×. (g–i) PVA-SA-Cellulose (1 g) beads: (g) externally surface at 25×, (h) cross-sectional views at 50× and (i) cross-sectional views at 200×.
