# Supplementary material for: Enhanced Durability of Cellulose-Reinforced PVA-SA Beads for Long-Term Quorum Quenching Applications in Membrane Bioreactors
Source: Gels. 2026 May 30;12(6):480. doi: 10.3390/gels12060480 (PMC13298359; doi:10.3390/gels12060480)
Supplement: Supplementary file 1 [file gels-12-00480-s001.zip › gels-4332099-supplementary.pdf]

Supplementary Notes (supporting information to the full text paper)

Enhanced Durability of Cellulose-Reinforced PVA-SA Beads for Long-Term Quorum Quenching Applications in Membrane Bioreactors

Noman Sohail [1]*, Thomas Fischer [2] and Marion Martienssen [1]

[1] Chair of Biotechnology of Water Treatment, Brandenburg University of Technology Cottbus-Senftenberg, Siemens-Halske-Ring 8, 03046 Cottbus, Germany; marion.martienssen@b-tu.de (M.M.)

[2] Central Analytical Laboratory, Brandenburg University of Technology Cottbus-Senftenberg, Siemens-Halske-Ring 8, 03046 Cottbus, Germany; thomas.fischer@b-tu.de (T.F.)

* Correspondence: noman.sohail@b-tu.de

Structural morphology of the beads using scanning electron microscopy:

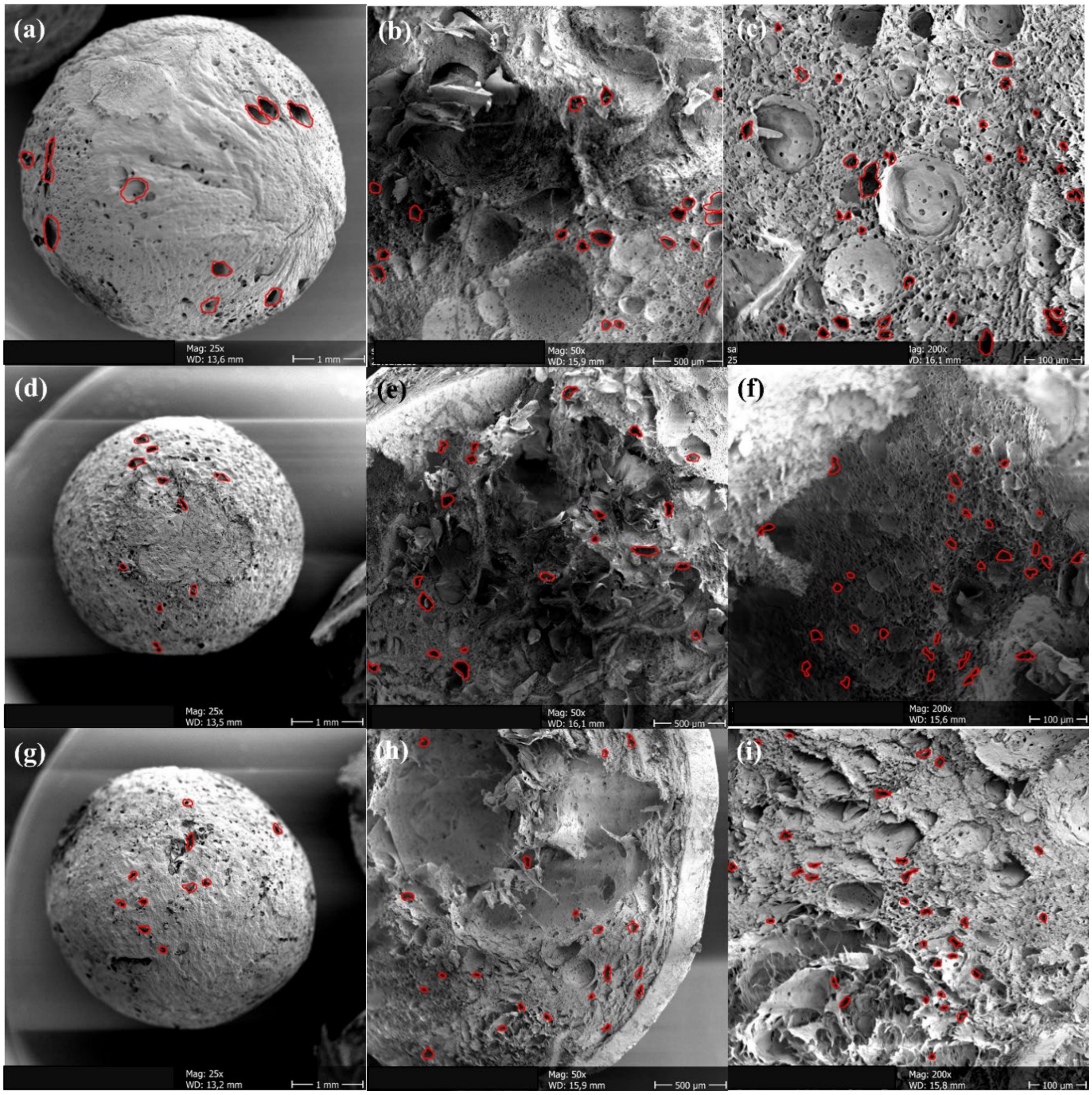

Figure S1. SEM images showing the external morphology and internal cross-sectional structures of the beads at different magnifications. (a-c) PVA-SA beads:

*Corresponding Author (Noman.sohail@b-tu.de)

(a) externally surface at 25×, (b) cross-sectional views at 50× and (c) cross-sectional views at 200×. (d-f) PVA-SA-Cellulose (0.5 g) beads: (d) externally surface at 25×, (e) cross-sectional views at 50× and (c) cross-sectional views at 200×. (g-i) PVA-SA-Cellulose (1 g) beads: (g) externally surface at 25×, (h) cross-sectional views at 50× and (i) cross-sectional views at 200×.

*Corresponding Author (Noman.sohail@b-tu.de)
